# Genomic diversity, population structure, and genome-wide association reveal genetic differentiation and trait improvements in mango

**DOI:** 10.1093/hr/uhae153

**Published:** 2024-07-01

**Authors:** Xiaowei Ma, Hongxia Wu, Bin Liu, Songbiao Wang, Yuehua Zhang, Muqing Su, Bin Zheng, Hongbing Pan, Bang Du, Jun Wang, Ping He, Qianfu Chen, Hong An, Wentian Xu, Xiang Luo

**Affiliations:** National Key Laboratory for Tropical Crop Breeding, Sanya 572024, China; Key Laboratory of Tropical Fruit Biology, Ministry of Agriculture & Rural Affairs; Key Laboratory for Postharvest Physiology and Technology of Tropical Horticultural Products of Hainan Province, South Subtropical Crops Research Institute, Chinese Academy of Tropical Agricultural Sciences, Zhanjiang, Guangdong 524091, China; National Key Laboratory for Tropical Crop Breeding, Sanya 572024, China; Key Laboratory of Tropical Fruit Biology, Ministry of Agriculture & Rural Affairs; Key Laboratory for Postharvest Physiology and Technology of Tropical Horticultural Products of Hainan Province, South Subtropical Crops Research Institute, Chinese Academy of Tropical Agricultural Sciences, Zhanjiang, Guangdong 524091, China; Panzhihua Academy of Agricultural and Forestry Sciences, Panzhihua, Sichuan 617061, China; National Key Laboratory for Tropical Crop Breeding, Sanya 572024, China; Key Laboratory of Tropical Fruit Biology, Ministry of Agriculture & Rural Affairs; Key Laboratory for Postharvest Physiology and Technology of Tropical Horticultural Products of Hainan Province, South Subtropical Crops Research Institute, Chinese Academy of Tropical Agricultural Sciences, Zhanjiang, Guangdong 524091, China; National Key Laboratory for Tropical Crop Breeding, Sanya 572024, China; Key Laboratory of Tropical Fruit Biology, Ministry of Agriculture & Rural Affairs; Key Laboratory for Postharvest Physiology and Technology of Tropical Horticultural Products of Hainan Province, South Subtropical Crops Research Institute, Chinese Academy of Tropical Agricultural Sciences, Zhanjiang, Guangdong 524091, China; National Key Laboratory for Tropical Crop Breeding, Sanya 572024, China; Key Laboratory of Tropical Fruit Biology, Ministry of Agriculture & Rural Affairs; Key Laboratory for Postharvest Physiology and Technology of Tropical Horticultural Products of Hainan Province, South Subtropical Crops Research Institute, Chinese Academy of Tropical Agricultural Sciences, Zhanjiang, Guangdong 524091, China; National Key Laboratory for Tropical Crop Breeding, Sanya 572024, China; Key Laboratory of Tropical Fruit Biology, Ministry of Agriculture & Rural Affairs; Key Laboratory for Postharvest Physiology and Technology of Tropical Horticultural Products of Hainan Province, South Subtropical Crops Research Institute, Chinese Academy of Tropical Agricultural Sciences, Zhanjiang, Guangdong 524091, China; Panzhihua Academy of Agricultural and Forestry Sciences, Panzhihua, Sichuan 617061, China; Panzhihua Academy of Agricultural and Forestry Sciences, Panzhihua, Sichuan 617061, China; Liangshan Academy of Forest and Grassland, Xichang, Sichuan 615000, China; Liangshan Academy of Forest and Grassland, Xichang, Sichuan 615000, China; Tropical Crops Genetic Resources Institute, Chinese Academy of Tropical Agricultural Sciences, Haikou, Hainan 571100, China; Bioinformatics and Analytics Core, University of Missouri, Columbia, MO 65201, USA; National Key Laboratory for Tropical Crop Breeding, Sanya 572024, China; Key Laboratory of Tropical Fruit Biology, Ministry of Agriculture & Rural Affairs; Key Laboratory for Postharvest Physiology and Technology of Tropical Horticultural Products of Hainan Province, South Subtropical Crops Research Institute, Chinese Academy of Tropical Agricultural Sciences, Zhanjiang, Guangdong 524091, China; College of Agriculture, Henan University, Zhengzhou, Henan 450046, China

## Abstract

Mango (*Mangifera indica* L.) has been widely cultivated as a culturally and economically significant fruit tree for roughly 4000 years. Despite its rich history, little is known about the crop’s domestication, genomic variation, and the genetic loci underlying agronomic traits. This study employs the whole-genome re-sequencing of 224 mango accessions sourced from 22 countries, with an average sequencing depth of 16.37×, to explore their genomic variation and diversity. Through phylogenomic analysis, *M. himalis* J.Y. Liang, a species grown in China, was reclassified into the cultivated mango group known as *M. indica*. Moreover, our investigation of mango population structure and differentiation revealed that Chinese accessions could be divided into two distinct gene pools, indicating the presence of independent genetic diversity ecotypes. By coupling genome-wide association studies with analyses of genotype variation patterns and expression patterns, we identified several candidate loci and dominant genotypes associated with mango flowering capability, fruit weight, and volatile compound production. In conclusion, our study offers valuable insights into the genetic differentiation of mango populations, paving the way for future agronomic improvements through genomic-assisted breeding.

## Introduction

Belonging to the Anacardiaceae family within the Sapindales order, the *Mangifera* genus encompasses approximately 70 species, with the cultivated mango (*Mangifera indica* L.) representing the most economically valuable member [1]. The plants’ edible fruit and diverse applications contribute to its status as the fifth largest fruit industry worldwide, with production following citrus, banana, grape, and apple [2, 3]. Presently, mango trees are grown in the tropical and subtropical regions of more than 100 countries between 30°S and 30°N, stretching from as far north as southern Sichuan in China and the southern islands of Japan, and as far south as southern Africa (http://www.fao.org/faostat). These regions maintain average temperatures above 11°C during the coldest months, with absolute minimum temperatures above −3.7°C. It is currently believed that cultivated mango was initially domesticated in the northern foothills of the India-Myanmar region before expanding into nearby Southeast Asia countries such as Thailand, Malaysia, Philippines, and Indonesia during the fourth and fifth centuries. Soon after, mango jumped to new continents, with cultivation occurring in Africa, South America, and North America [4, 5]. The evolutionary process, encompassing both natural genetic progression and conscious human intervention, has yielded a broad spectrum of phenotypic mango diversity. Variability can be observed in fruit shape, size, color, taste, flower type, and various other agronomic traits [4, 6–8]. For instance, fruit shapes can be more round, ovate, or oblong, and their weights can span from less than 50 g to over 2000 g. Flesh colors vary from milky white to yellow to orange, and textures range from smooth to fibrous. Diversity is also evident in seed structures, which may be either monoembryonic or polyembryonic. Characterizing the genetic variations associated with valuable agronomic traits and gaining a deeper understanding of the genomic diversity and domestication history of mango are pivotal for future crop improvement endeavors.

Mango domestication is marked by a series of crosses and continuous human selection; however, the use of hybridization to enhance traits in mangoes is challenged by the fruits’ natural inclination to self-pollinate. Mango flowers blossom on terminal inflorescences, which are generally conical and reach up to 60 cm depending on the variety. Each inflorescence bears roughly 4000 individual flowers, with each measuring between five and ten mm. Despite this, mangoes have an average fruit set of less than 3% in open pollination conditions. While hand-pollination may show slight improvement, the method is impractical due to its low efficiency [9, 10], and difficulties imposed on constructing a genetic population for quantitative trait loci (QTL) analyses. Previous research has endeavored to unveil mango genetic diversity using an array of marker types such as simple sequence repeats (SSRs) [11], random amplified polymorphic DNAs (RAPD) [12], amplified fragment length polymorphisms (AFLP) [13], chloroplast inter simple sequence repeat (cpISSR) [14], and restriction site associated DNA sequencing [4]. However, the small population sizes and limited number of markers have restricted the impact of these studies. Additionally, no gene editing system has been established for the crop, hindering functional genomic progress and limiting breeding advancements.

Recently, advancements in genome re-sequencing have allowed researchers to explore genetic population diversity in both wild and cultivated woody perennial fruit crops, such as walnut [15], peach [16], pears [17], and citrus [18]. This facilitates the exploration of evolutionary history and enables informed molecular crop improvements going forward. The release of chromosome-scale genome assemblies of mango cultivars such as ‘Hong Xiang Ya’ [19], ‘Alphonso’ [2], ‘Tommy Atkins’ [20], and ‘Irwin’ [21] have proven beneficial in comprehending the origin and evolutionary history, and enabled the identification of genetic loci associated with phenotypic traits such as fruit quality and yield. Large-scale population and genotype–phenotype association studies have been instrumental in unveiling the emergence of important agronomic traits in various crops [22–24]. In the present study, we collected and re-sequenced samples from 224 mango accessions across 22 countries, encompassing relative species, landrace, and cultivated genotypes. We then performed a genome-wide association study (GWAS) to scrutinize the genetic basis underlying flowering capability, fruit weight, and aroma-related volatile compounds. Our results further elucidate the phylogenetic relationships among mango ecological groups, laying a theoretical foundation for future crop enhancement and the effective use of germplasm resources.

## Results

### Re-sequencing germplasm resources revealed a vast amount of SNPs

**Figure 1:**
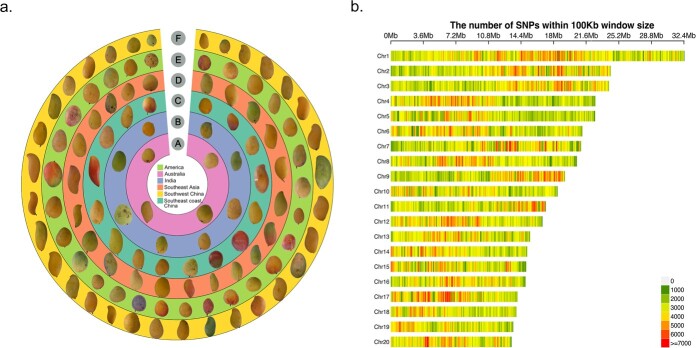
Fruit features and genome-wide variant calling of mango accessions. **a** Representative fruits sampled from various geographical regions. **b** Chromosomal distribution of identified SNPs.

In this study, we re-sequenced 224 diverse mango germplasm samples (Table S1, see online supplementary material), consisting of 220 *M. indica* accessions and four purportedly related species within the *Mangifera* genus (one *Mangifera odorata* Griff., one *Mangifera himalis* J.Y. Liang, and two *Mangifera persiciformis* Wu & Ming). These accessions were collected from diverse eco-geographic regions, including India, Southeast Asia, China, Africa, South America, the United States of America, the Caribbean, Australia, Mexico, Sri Lanka, and Israel (Fig. 1a; Table S1, see online supplementary material). In total, we generated 1.5 Tb clean reads after filtering, with each sample contributing roughly 6.5 GB of data. This is equivalent to covering the ~392.9 Mb mango genome [2] approximately 16.37 times. The mapping rate for 220 of the accessions ranged from 94.99% to 99.30%, with a mean of ~98.81% (Table S2, see online supplementary material).

Using the genome analysis toolkit (GATK) [25], we identified a total of 12 473 629 single nucleotide polymorphisms (SNPs) unevenly distributed across the 20 mango chromosomes (Fig. 1b). Chromosome 1 was the most populated, with a total of 1 122 510 SNPs and an average density of 34.6 SNPs per kb. In contrast, chromosome 19 had the fewest, with 440 664 SNPs and an average density of 32.6 SNPs per kb (Table S3, see online supplementary material).

Our variant detection analysis identified a total of 25 211 317 SNPs, among which 38.42% (9685306) were within intergenic regions, 24.26% (6116625) were within 5 Kbp downstream of transcripts, 25.60% (6453044) were within 5 Kbp upstream of transcripts, 8.20% (2067221) were within introns, and 3.53% (889121) were within exons. Of the SNPs located in the transcript region, 40.17% (357133) were synonymous and 57.83% (514168) were missense (Table S4, see online supplementary material).

### Mango population structures are consistent with geographic distribution

**Figure 2:**
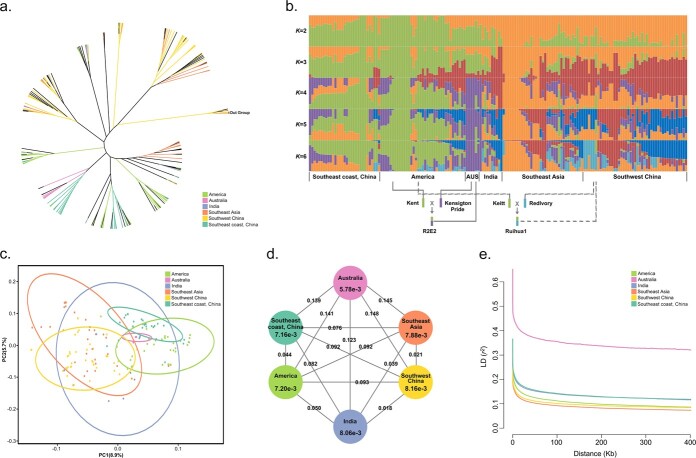
Genetic structure of mango based on 224 examined accessions. **a** Neighbor-joining phylogenetic tree based on SNPs. Branch colors indicate different subgroups. **b** Population structures inferred using admixture analysis for an assumed number of groups (K) from 2 to 6. Two documented modern breeding events are indicated below the plot. **c** PCA of the first two components of all accessions. **d** Nucleotide diversity (θπ) and population divergence (*Fst*) across all groups. Each group is annotated with the nucleotide diversity estimation. Values along the lines indicate the pairwise population divergence. **e** LD decay for each group.

To elucidate the genetic diversity of the 224 sampled accessions, we utilized high-quality SNPs to create a phylogenetic tree (Fig. 2a; Fig. S1, see online supplementary material) and conducted both admixture (Fig. 2b) and principal component (PCA) (Fig. 2c) analyses. The phylogenetic tree revealed an outgroup cluster comprising *M. odorata* Griff. and *M. persiciformis* Wu & Ming, indicating they are not members of *M. indica.* This finding is consistent with differences observed during morphological classification [3, 14]. However, *M. himalis* J.Y. Liang was intertwined with the *M. indica* accessions, suggesting it may be a sibling genotype of *M. indica* instead of a separate species.

Except for *M. himalis* J.Y. Liang, all other accessions could be divided into either Clade I or Clade II, aligning with their geographical origins (Fig. 2a). Accessions from India were grouped into Clade I and accessions from Southeast Asia were grouped into Clade II, indicating two predominant centers of origin [1]. These findings are based on an admixture analysis, using 0.5 as the probability of membership threshold (Fig. 2b). When K = 3, Clade I was further divided into two sub-populations: Pop 1, comprising accessions from India and Southwest China; and Pop 2, encompassing accessions from Southeast Asia and Southwest China (Fig. 2b). At K = 4, most Australian mango accessions separated from the American ones in Clade II (Fig. 2b). Finally, at K = 5, most accessions of the Southeast coastal region of China (SCRC) also diverged from American ones (Fig. 2b). Notably, there was little change within the American mango population structure with increasing K values supporting the assertion that the elevated uniformity within the group emerged as a result of shaping under a singular environment [5]. It is also possible that the American accessions represent a more modern cultivar, as hybrid breeding techniques in this country date back to the 19th century [4, 5]. Our PCA largely reflected the population structure results (Fig. 2c). Collectively, six subpopulations—India, Southeast Asia, America, Southwest China, SCRC, and Australia—could explain the structure of our accession panel, aligning with the geographical origin of these germplasms.

The population structure inferred from K = 4 indicated a cross-breeding event, in which ‘R2E2’ was established from a hybridization of ‘KensigtonPride’ (Australian cultivar) and ‘Kent’ (American cultivar) [5]. Similarly, ‘Ruihua 1’ (Southwest Chinese cultivar) was determined to be the descendant of ‘Keitt’ (American cultivar) × ‘RedIvory’ (Southwest Chinese cultivar) (Fig. 2b). This hybrid breeding history further supports the population structure constructed with extensive SNP data.

### Differentiation and genetic population diversity of Chinese mango

Between all phylogenetic subpopulations, the nucleotide diversity (θπ) ranged from 5.78 × 10^−3^ to 8.16 × 10^−3^ and the pairwise *Fst* ranged from 0.018 to 0.148 (Fig. 2d), indicating relatively low differentiation. A gradual decrease in θπ was detected from Southwest China, India, Southeast Asia, America, SCRC, and Australia. This reflects the genetic differentiation caused by domestication, a series of crosses, and continuous human selection [4, 8].

Additionally, there was a 0.021 *Fst* between Southwestern Chinese and Southeast Asian accessions and a 0.018 *Fst* between Southwestern Chinese and Indian accessions. While the *Fst* between Southwestern Chinese and American accessions was 0.093, those from Southwestern China reflected a higher θπ and a faster linkage disequilibrium (LD) decay compared to those from America (Fig. 2e). Our results suggest that Southwestern Chinese mangos were primarily introduced or received gene flow from wild mangos in Southeast Asia or India. Further comparisons revealed that the *Fst* between SCRC and Southwestern China accessions was 0.092, which is significantly greater than the 0.044 *Fst* observed between SCRC and American accessions. These results indicate that SCRC and American accessions share a relatively close genetic relationship, further supporting our constructed phylogenetic tree (Fig. 2a).

### GWAS of flowering capability

**Figure 3:**
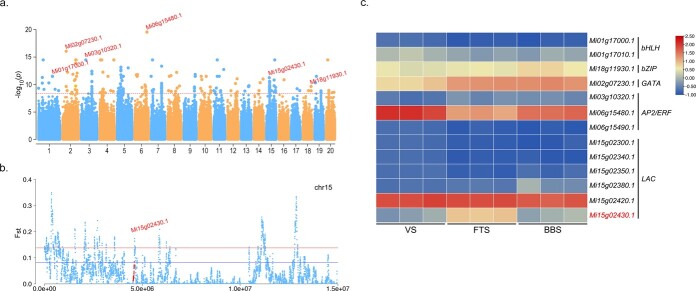
**a** Manhattan plot of flowering capability GWAS. The red line indicates associated SNP significance with a threshold value of -log10P ≥8. Strongly associated SNPs and candidate genes are annotated. **b** Association signals of GWAS and *Fst* in significant signal regions. **c** Expression patterns of candidate genes in buds during three growth stages based on estimated FPKM. BBS, bud break stage; FTS, floral transition stage; VS, vegetative stage.

Flowering capability is tightly correlated with fruit yield, making it an essential trait in mango. The collected accessions demonstrated a wide variation of flowering capability (Table S5, see online supplementary material), satisfying the conditions necessary to detect genomic loci associated with the trait through a GWAS. A total of 12 significantly associated SNPs were identified and distributed across eight chromosomes (1, 2, 3, 4, 6, 10, 15, and 18). Within the corresponding associated regions, 174 annotated protein-coding genes were located (Fig. 3a; Tables S6 and S7, see online supplementary material). Several of these genes had previously been reported to regulate flower development processes, including transcription factors *bHLH* (*Mi01g17000.1* and *Mi01g17010.1*), *bZIP* (*Mi18g11930.1*), *GATA* (*Mi02g07230.1*), and *AP2*/*ERF* (*Mi03g10320.1*, *Mi06g15480.1*, and *Mi06g15490.1*) [26–29]. These previous reports confirm the validity of our GWAS results. Additionally, six genes on chromosome 15—*Mi15g02300.1*, *Mi15g02340.1*, *Mi15g02350.1*, *Mi15g02380.1*, *Mi15g02420.1*, and *Mi15g02430.1*—belong to the laccase (LAC) family, which is associated with flowering ability (Table S7, see online supplementary material). These genes were annotated to play important roles in plant physiological and biochemical processes [26, 27]. Our RNA-seq analysis demonstrated that the expression levels of *Mi18g11930.1*, *Mi02g07230.1*, *Mi03g10320.1, Mi15g02350.1*, *Mi15g02380.1*, and *Mi15g02430.1* were significantly up-regulated, while the expression of *Mi01g17010.1*, *Mi06g15480.1,* and *Mi15g02420.1* were remarkably down-regulated in buds during the transition from vegetative growth to reproductive development (Fig. 3c; Table S8, see online supplementary material). These genes may be differentially expressed to affect flowering ability. For instance, *Mi15g02430.1* on chromosome 15 was identified as a selective sweep region based on the top 5% *Fst* values, suggesting its potential selection footprint during breeding efforts aimed at improving flowering capability (Fig. 3b).

### GWAS of fruit weight

**Figure 4:**
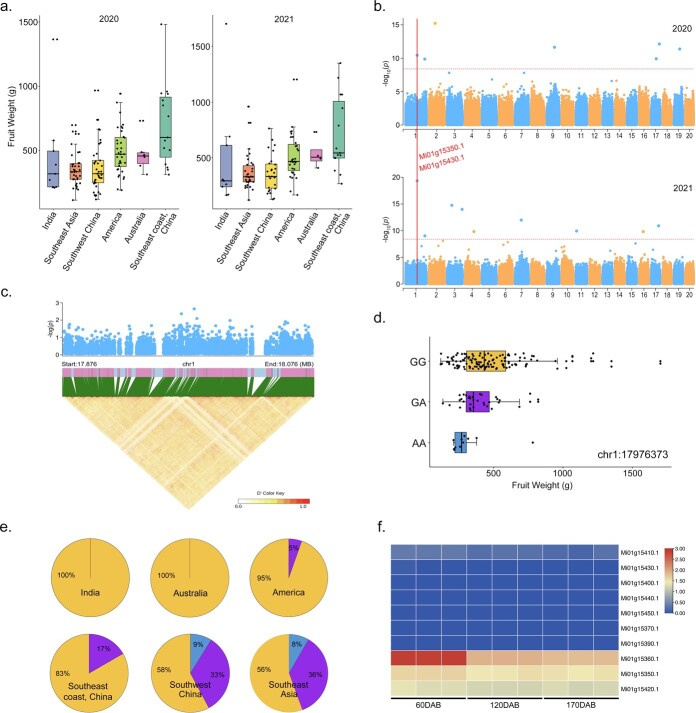
**a** Average fruit weights between mango groups. **b** Manhattan plot of fruit weight GWAS. **c** Chromosome 1 (bottom panel) LD heat map of the candidate region between 17.876 and 18.076 Mb. **d** Fruit weight based on the three genotypes (Gen.). **e** Proportion of genotypes across mango groups. **f** Effect of candidate gene expression patterns on fruit weight at various fruit development stages in cultivar ‘Keitt’. DAB, days after bloom.

Fruit weight is a highly valued agronomic trait that is essential in mango breeding and domestication. Our accessions displayed wide variations in fruit weight, ranging from 117.20 g to 1703.33 g (Table S5, see online supplementary material). We observed that accessions from America had notably larger fruit weights when compared with those from other countries. This suggests that fruit weight may endure intensive selection in America (Fig. 4a). The mango industry in America has given rise to many popular commercial varieties, many of which are renowned for their elite yields [4, 5]. This reflects the increasing emphasis on American production, which continues to serve as a crucial foundation for modern mango breeding worldwide.

We next performed a GWAS to identify variants associated with fruit weight and identified one significant signal spanning a 17.87–18.07 Mb interval on chromosome 1 (Fig. 4b and c; Table S9, see online supplementary material). As a result, we identified a total of 11 candidate genes in the associated region (*Mi01g15350.1*, *Mi01g15360.1*, *Mi01g15370.1*, *Mi01g15380.1*, *Mi01g15390.1*, *Mi01g15400.1*, *Mi01g15410.1*, *Mi01g15420.1*, *Mi01g15430.1*, *Mi01g15440.1*, and *Mi01g15450.1*) (Table S10, see online supplementary material). Among these, *Mi01g15430.1* had previously been found to encode a member of the epidermal patterning factor-like family (EPFL1). This family is primarily involved in *Arabidopsis* [30] and rice [31]. *Mi01g15350.1* is orthologous to *AT5G10350 in Arabidopsis*, whose differential expression is believed to regulate cell expansion, thereby increasing petal size [32]. Our RNA-seq analysis for fruit flesh samples collected from the cultivar ‘Keitt’ (American accession) showed a gradual decrease in the expression levels of *Mi01g15350.1*, which contrasted the simultaneous increase in fruit weight during development (Fig. 4f; Fig. S2, see online supplementary material).

Additionally, the peak SNP (chr1: 17976373) generated three genotypes, Gen.1 (AA), Gen.2 (GA), and Gen.3 (GG). Accessions harboring the GG genotype exhibited significantly higher fruit weights than those with the heterozygous alleles, suggesting the homozygous Gen.3 is the dominantly contributing genotype (Fig. 4d). Gen.3 was mainly detected in model cultivars originating from America, Australia, and SCRC, indicating intensive yield trait selection during breeding processes (Fig. 4e).

### GWAS of fruit volatile compounds

A total of 15 characteristic compounds were identified from 209 mango accessions in two years, including eight monoterpenes, three sesquiterpenes, two non-terpene hydrocarbons, and two aldehydes (Table S5, see online supplementary material). We noted substantial variations in the volatile compound contents between populations (Fig. S3, see online supplementary material). Notably, American mango cultivars have accumulated fewer volatile compounds (15 total) than Southeast Asian and Indian accessions, indicating that volatile compounds may have been selected against in modern American breeding.

**Figure 5:**
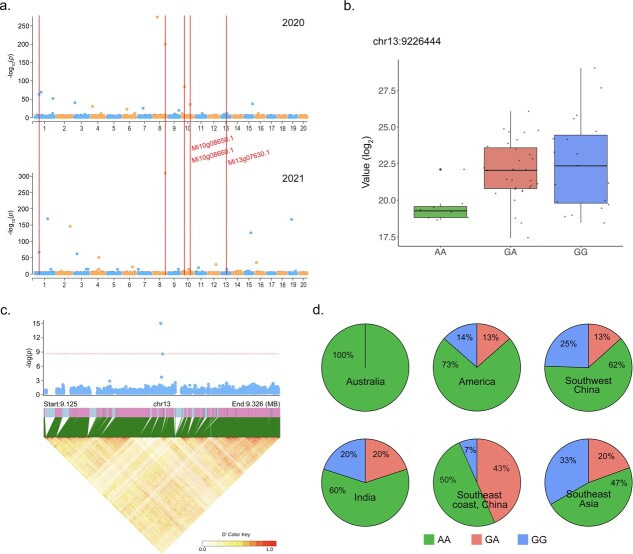
**a** Manhattan plot of β-Ocimene content GWAS. **b** β-Ocimene content based on the three established genotypes (Gen.). **c** Chromosome 13 (bottom panel) LD decay plot of the candidate region between 125 and 9.326 Mb. **d** Proportion of genotypes across mango groups.

Our GWAS consistently identified 12 SNPs that were significantly associated with six of the 15 volatile compounds sampled over both years (Table S9, see online supplementary material). Additionally, β-Ocimene content was significantly associated with four SNPs identified on chromosomes 1, 8, 10, and 13. In total, 106 genes were observed (Fig. 5a;Table S10, see online supplementary material). A particularly interesting gene named *Mi13g07630.1* was annotated around 31 kb upstream of the peak SNP (chr13: 9226444) on chromosome 13 (Fig. 5c). This gene is likely involved in the formation of volatile compounds, as it belongs to the ERF transcription factor gene family, which is known to play a role in terpenoid biosynthesis [33]. The peak SNP (chr13: 9226444) generated three genotypes—Gen.1 (AA), Gen.2 (GA), and Gen.3 (GG)—the latter of which is correlated with a higher β-Ocimene content (Fig. 5b). Further analyses revealed that Southeast Asian and Southwestern Chinese accessions displayed a higher frequency of the GG alleles compared to modern cultivars from America, Australia, and SCRC, which contained a higher frequency of the heterozygous GA alleles (Fig. 5d). Taken together, these results suggest that the emphasis on yield traits in American cultivars may lead to a reduction in fruit aroma. On chromosome 10, we selected a total of seven candidate genes for further investigation, including *Mi10g08640.1*, *Mi10g08650.1*, *Mi10g08660.1*, *Mi10g08670.1*, *Mi10g08680.1*, *Mi10g08690.1*, and *Mi10g08700.1*. An RNA-seq analysis revealed that during fruit maturation, the expression levels of *Mi10g08650.1* and *Mi10g08660.1* increased, while *Mi10g08670.1*, *Mi10g08680.1*, *Mi10g08690.1*, and *Mi13g07630.1* decreased. This finding gives us a glimpse of the complex network regulating β-Ocimene accumulation (Table S11 and Fig. S5, see online supplementary material).

For 1R-α-Pinene content, singular significantly associated SNPs were identified on chromosomes 1 and 3. Following annotations of both SNPs, we obtained nearby 56 genes, 20 of which were distributed across the 28.95 to 29.15 Mb region of chromosome 1 (Fig. S4a, Tables S9 and S10, see online supplementary material). An additional RNA-seq analysis showed that during fruit maturation, *Mi01g25080.1*, *Mi03g04820.1*, and *Mi03g04950.1* were gradually upregulated, while *Mi03g04720.1*, *Mi03g04730.1*, *Mi03g04940.1*, and *Mi03g05040.1* were gradually downregulated (Table S11, see online supplementary material). These expression patterns may be associated with the accumulation of 1R-α-Pinene (Fig. S5, see online supplementary material).

Only one SNP in the region from 18.02 to 18.22 Mb on chromosome 5 was associated with Neo-Alloocimene content, and we obtained 29 surrounding genes (Fig. S4b, Tables S9 and S10, see online supplementary material). During fruit maturation, our RNA-seq analysis illustrated a gradual decrease in the transcription levels of *Mi05g16830.1*, *Mi05g16870.1*, *Mi05g17020.1*, and *Mi05g17050.1*, which reflects the trend of Neo-Alloocimene accumulation (Table S11 and Fig. S5, see online supplementary material).

One SNP in the region from 9.87 to 10.07 Mb of chromosome 16 was significantly related to caryophyllene content, and we obtained six genes following annotation (Fig. S4c, Tables S9 and S10, see online supplementary material). During fruit development and ripening, our RNA-seq indicated a gradual increase in the transcription levels of *Mi16g08430.1* and *Mi16g08440.1*, which both encode ABC transporters (Table S11, see online supplementary material).

We identified singular SNPs on chromosomes 4 and 7, which were significantly associated with β-Myrcene content. We next predicted 40 genes (Fig. S4d and Tables S9 and S10, see online supplementary material), among which, *Mi04g06120.1*, *Mi04g06140.1*, *Mi04g06150.1*, and *Mi04g06160.1* (located on chromosome 4) were identified under selection. An RNA-seq demonstrated a down-regulation of *Mi04g06120.1*, *Mi04g06150.1*, and *Mi04g06160.1* and an up-regulation of *Mi07g10670.1*, which encodes a caseinolytic protease, during mango maturation (Table S11, see online supplementary material).

Finally, one SNP located in the region from 28.95 to 29.15 Mb on chromosome 1 was associated with D-limonene content, and 20 genes were predicted (Fig. S4e and Tables S9 and S10, see online supplementary material). Among these, *Mi01g25060.1* functions as terpene synthase and biosynthesizes volatile terpenes. The transcription levels of *Mi01g25070.1* were found to gradually decrease during mango fruit maturation, which reflects the D-limonene accumulation trend (Table S11and Fig. S5, see online supplementary material).

## Discussion

Genomic variations in mango trees offer a valuable resource for the exploration of genetic diversity, gene mapping, selection, and molecular-assisted breeding. Previous studies of mango genetic diversity predominantly focused on a limited amount of restriction site-associated DNA markers and very few mango accessions [12–14]. Previously, a total of 6594 AFLP markers were employed to construct a genetic linkage map based on 173 F_1_ mangoes obtained from a cross between ‘Jin-Hwang’ and ‘Irwin’ [34]. Additionally, eight AFLP primer combinations were employed to categorize 35 accessions into four groups corresponding with the different *Mangifera* species. Recently, researchers revealed that Indian and Southeast Asian accessions constitute two diversity centers of cultivated mango. Here, we assessed the genetic variation of 224 mangos collected from diverse geographical regions, with a particular focus on Chinese accessions, to examine their classification and genetic differentiation. Our phylogenomic analysis strongly supports the reclassification of *M. himalis* J.Y. Liang, which is exclusively grown in China, as *M. indica* (cultivated mango). This suggestion conflicts with traditional morphological taxonomy, which had previously labeled *M. himalis* J.Y. Liang as a separate species within the Mangifera genus.

Our integrated analyses, which include assessments of genetic diversity, phylogenetics, population structure, and PCA, indicated that our accessions could be categorized into six distinct subpopulations: India, Southeast Asia, Southwest China, America, SCRC, and Australia. Notably, accessions from India, Southeast Asia, and Southwest China displayed a higher genetic diversity than those from America. This suggests that American-cultivated mangoes may possess a narrower genetic basis, which may be attributed to prolonged selective breeding processes. This diminished genetic diversity is likely caused by an increase in homozygous sites (chr1: 17976373 (GG)) associated with fruit weight, which began accumulating during the 19th century when extensive crop improvement projects were initiated in America. In contrast, our data hints at the ancient cultivation history of Chinese mangos, which may be traced back to the Ming Dynasty around 1545 [35]. The geographic proximity of Southwest China to the primary *M. indica* diversity center in Southeast Asia [4, 5] indicates the possibility of gene flow or accession exchange between the two regions. Moreover, our phylogenetic analysis demonstrated domesticated mango accessions from Southwest China intermingling with those from Southeast Asia, indicating a shared lineage. Lastly, accessions from Southwest China exhibit a higher diversity than those from India and Southeast Asia, reflecting the presence of wild characteristics.

Chinese mango accessions can be divided into two prominent geographic clusters: Southwest China and SCRC, each showcasing distinctive ecological characteristics. The low *Fst* values along with the close phylogenetic relationships between accessions from Southwest China, Southeast Asia, and India strongly imply that accessions from Southwest China were domesticated from lineages originating in Southeast Asia and India. This assertion is supported by the presence of mango relatives and ancient mango trees (approximately 1000 years old) flourishing in the limestone mountainous regions bordering Guangxi and Yunnan provinces in China. The dispersal of mangoes from India and Southeast Asia to Southwest China likely coincides with the spread of Buddhism or Dai ethnic folk culture. Deeply rooted in Theravada Buddhism, the Yunnan Dai ethnic settlement areas form part of a larger Dai language region, which includes northeast India, central northern Myanmar, northern Thailand, northern Laos, northwest Cambodia, northwest Vietnam, and western Yunnan [35]. This movement corroborates various historical records, which indicate that mango was introduced to Southeast Asia through Buddhist monks around the fourth century [4]. Conversely, intermingling in our phylogenetic analysis suggests that accessions of SCRC may have been introduced from America. This inference is supported by the higher genetic similarity in their genomes compared with the accessions from other regions. Despite this genetic similarity, the population structures suggest that there is no introgression between the two populations. Thus, the intermixing of the two populations likely stems from recurrent commercial trades or accession exchanges between SCRC and America.

It is well-known that artificial selection and domestication have significantly altered the quality traits of wild fruit crop progenitors [36–38]. For instance, the domestication of mango has resulted in increased fruit size, enhanced sweetness, vibrant coloration, and reduced flesh acidity and fibrousness [1]. Identifying the genes associated with these traits is crucial for ongoing crop improvements involving molecular breeding. Although five mango genetic linkage maps have been published [34, 39–42], there has been little progress in mapping and cloning the functional genes or major QTLs contributing to consumer-driven traits [34]. In this study, we first conducted a GWAS of mango accessions which revealed 26 loci associated with flowering ability, fruit weight, and fruit aromatic compounds. We next predicted 422 genes associated with these loci. The loci influencing fruit weight appear to be distinct from those affecting aroma, implying that elite cultivars with both high yield and quality can be achieved through hybrid breeding or genetic editing. Among the identified genes, several candidates are associated with flowering capability, fruit weight, and volatile compound composition. Typically, certain transcription factors such as bHLH, bZIP, GATA, AP2/ERF, and laccase (LAC) family genes are known to regulate flower development processes, thereby affecting flowering capability. *Mi01g15430.1* was identified as a bHLH transcription factor that negatively regulates petal size, suggesting that it influences mango fruit weight. Furthermore, encoding an ERF transcription factor, *Mi13g07630.1* may have the potential to influence the accumulation of β-Ocimene content by controlling terpenoid biosynthesis, thereby affecting aroma. Homologous genes performing similar functions have been previously reported in sweet orange [33], *Litsea cubeba* [43], and maize [44]. Together, these findings significantly advance our understanding of the mechanisms governing valuable mango breeding traits and provide valuable candidate genes for further functional research.

Our observations reveal significant insights into the mango breeding and domestication process. First, we observed that the dominant genotypes associated with fruit weight (chr1: 17976373 (GG)) and aroma (chr13: 9226444 (GG)) were homozygous, indicating that the improvement of these traits relies on accumulated effects rather than heterosis. Second, the dominant fruit weight genotypes were most prevalent in accessions from America, Australia, and SCRC, which also had a distinct absence of dominant aroma-related genotypes. Phenotypically, accessions from these regions showcase larger fruit weights but lower accumulations of volatile substances compared to those from Southeast Asia, India, and Southwest China. Similar phenomena have been reported in other fruit crops like peaches [16], apples [45], and pears [17]. Moreover, we elucidated the selection pressures influencing several genes associated with flowering capability and fruit terpenoid content, shedding light on the intricate dynamics of mango domestication.

## Conclusion

This study presents a high-resolution genomic variation map developed from a substantial number of samples, offering novel insights into mango classification, population diversity, and domestication. Additionally, we identified several candidate genes associated with highly sought-after traits such as flowering capability, fruit weight, and volatile compounds. These genes may serve as valuable tools in methods like molecular-assisted breeding, enabling the improvement of future mango cultivars. Furthermore, we explore several significant dominant genotypes, their distribution, and their impact on specific breeding traits, providing a clarified portrayal of the selection and domestication dynamics involved in mango breeding.

## Materials and methods

### Plant materials

A total of 220 mango (*M. indica* L.) accessions were collected from various regions across the globe. Additionally, one *M. odorata* Griff., and one *M. himalis* J.Y. Liang were collected from Baoshan, Yunnan, China, and two *M. persiciformis* Wu & Ming were collected from Baise, Guangxi, China. The scions of 220 accession were obtained from the mango breeding and preservation unit, then grafted onto a rootstock ‘Tumang’ and planted in the South Subtropical Crops Research Institute (SSCRI) mango field (E110^°^4′, N21^°^12′) in Mazhang County, Guangdong, China. Young, healthy, and fresh leaves were sampled from each accession in 2019 and then stored at −70°C. Genomic DNA was extracted using the improved cetyltrimethylammonium bromide (CTAB) method [46], and the quality and quantity was evaluated with the NanoDrop™ One spectrophotometer (Thermo Fisher Scientific, Waltham, MA, USA).

The RNA-Seq analysis utilized apical buds from one-year-old branches of mango (*M. indica* L. Tainong 1) sampled at the vegetative stage (VS), the floral transition stage (FTS), and the bud break stage (BBS). Mango (*M. indica* L. Keitt) fruits were harvested at 60, 150, 189, and 199 (fully ripe) days after bloom (DAB). Fruit weights were measured with an electronic scale. Pulp was manually separated from fruit samples, immediately diced frozen in liquid nitrogen, and then stored at −70°C for volatile content and RNA-Seq analysis. Each stage was sampled with three biological replicates, each of which consisted of the pooled flesh from six fruits.

### DNA sequencing, SNP calling, and functional annotation

A minimum of 4 μg genomic DNA was used to construct all sequencing libraries following the manufacturer’s instructions (Illumina Inc., San Diego, CA, USA). The libraries were sequenced with a Hiseq 2500 sequencer with rapid-run mode, generating 250-bp paired-end reads. Over 5 GB of sequence data was generated for each sample. NGSQCToolkit (version 2.3.3) software [47] was employed to remove paired-end reads containing adapter sequences and low-quality reads (read with >30% bases and Phred quality ≤25). After filtering, the clean reads of all accessions were aligned to the mango reference genome (*M. indica* L. cv. Alphonso) with Burrows–Wheeler Aligner (BWA, version 0.7.12) software [48]. The mapped reads were then sorted with Samtools and SNP calling was performed using the Genome Analysis Toolkit (GATK, version 4.4.0.0) [49]. The raw SNPs were hard filtered with the following parameters: ‘QD < 2.0 || MQ < 40.0 || FS > 60.0 || SOR > 3.0 || MQRankSum < −12.5 || ReadPosRankSum < −8.0’. Based on the mango reference genome, identified SNPs were further annotated using the package ANNOVAR (version: 2015-12-14) [25].

### Population genetics and linkage disequilibrium analysis

FigTree 1.4.3 (http://tree.bio.ed.ac.uk/software/figtree/) was employed to create a neighbor-joining tree with the accessions. The population structure was inferred using ADMIXTURE (version 1.3) [50] with *K* values ranging from 2 to 6. A PCA was conducted and the first two eigenvectors were plotted using the R package poppr [51] with default parameters. Nucleotide diversity (π) and pairwise genetic differentiation (*Fst*) in a 100-kb sliding window with a 10-kb step were calculated using VCFtools (version 0.1.14) [52]. LD decay was calculated for each population using the PopLDdecay package (version 3.4) with the parameters −*MAF* 0.05 − *Het* 0.88 − *Miss* 0.25 − *MaxDist* 300 [53].

### Evaluation of fruit weight and flowering capability

Fruits from each accession were sampled in two successive years (2020–2021) once they reached commercial maturity based on skin color, soft touch, and aroma. During the growing period, all accessions were equally managed. Once harvested, sampled fruits were washed, drained, and dried with paper towels. Maximum fruit weight was determined by weighing at least 15 fruits from each accession.

Flowering capability was defined as a qualitative trait and is reflected by the flowering rate. During the full blossoming period from January to March 2020 and 2021, 100 one-year branches were randomly collected from three trees from each accession. Flowering rate (%) = number of branches with flowers/total number of selected branches × 100%. If the flowering rate was less than 30%, the accession was recorded as ‘1’ for low flowering capability. If the flowering rate was 30% to 80%, the accession was recorded as ‘0’ for medium flowering capability. If the flowering rate was more than 80%, the accession was rated as ‘2’ for high flowering capability.

### Measurement of fruit volatile content

Volatile compound contents of mango fruit were evaluated in 2020 and 2021. The extraction and measurement of volatiles were carried out based on the previous methods [54]. A mixture of frozen flesh powder (2 g) and six mangoes was transferred into a 20 ml vial containing 5 ml saturated NaCl solution and 30 μl internal standard (0.29 mg/L Nonanoic acid, methyl ester). The vials were transferred to an ultrasonic cleaner model and heated at 40°C for 10 min. Volatiles were extracted using a solid-phase microextraction (SPME) fiber coated with 65 μm polydimethylsiloxane-divinylbenzene (Supelco, Bellefonte, PA, USA), then desorbed at the injection for 3 min at 240°C. The contents of the volatiles were measured using an Agilent gas chromatograph model 6890 N coupled with an Agilent 5973 N mass selective detector (Agilent, Santa Clara, CA, USA). The initial oven temperature was 40°C for 5 min, before being raised to 70°C at 2°C/min, for 2 min, then to 120°C at 3°C/min, then to 150°C at 5°C/min, and finally to 220°C at 10°C/min, for 2 min. The transfer temperature, ion source temperature, and electron ionizations were 250°C, 230°C, and 70 eV, respectively. Volatiles were identified using the database of the NIST Mass Spectral Library (NIST-08), and the quantitative content was calculated with the peak of the internal standard as a reference.

### Genome-wide association analysis

We conducted a GWAS for flowering capability using a Factored Spectrally Transformed Linear Mixed Model (FaST-LMM) [55]. Other traits including fruit weight, 1R-α-Pinene, Neo-Alloocimene, Caryophyllene, β-Myrcene, and D-Limonene were analysed using fixed and random model circulating probability unification (FarmCPU) [56] based on high-quality SNPs. A Bonferroni correction threshold was applied (*P*-value = 0.1/marker number) to identify significant associations between SNPs and traits. Candidate genes were screened within a region spanning 200 kb upstream and downstream of the associated signals, using the mango ‘Alphonso’ genome as a reference [2]. The LD heatmap was generated and visualized using LDheatmap (version 1.40) [57].

### Transcriptome sequencing and analysis

The mRNA of all samples was extracted using a Huayueyang Plant RNA Extraction Kit (Quick RNA isolation Kit; Haidian District, Beijing). Library construction was completed using the Illumina HiSeq™ 2000 platform (Illumina). After removing the adapter and low-quality reads with Trimmomatic version 0.33 [58], the TopHat (version 2.1.0) [58] was used to align the paired-end clean reads to the mango genome [2]. Gene expression levels were calculated using the value of fragments per kilobase of transcript per million fragments mapped (FPKM) with RSEM (version 1.2.15) [59].

## Supplementary Material

Web_Material_uhae153
