# Supplementary material for: Genomic diversity, population structure, and genome-wide association reveal genetic differentiation and trait improvements in mango
Source: Hortic Res. 2024 Jul 1;11(7):uhae153. doi: 10.1093/hr/uhae153 (PMC11246242; doi:10.1093/hr/uhae153)
Supplement: Web_Material_uhae153 [file web_material_uhae153.zip › supplementary_figures_ S1-S5.docx]

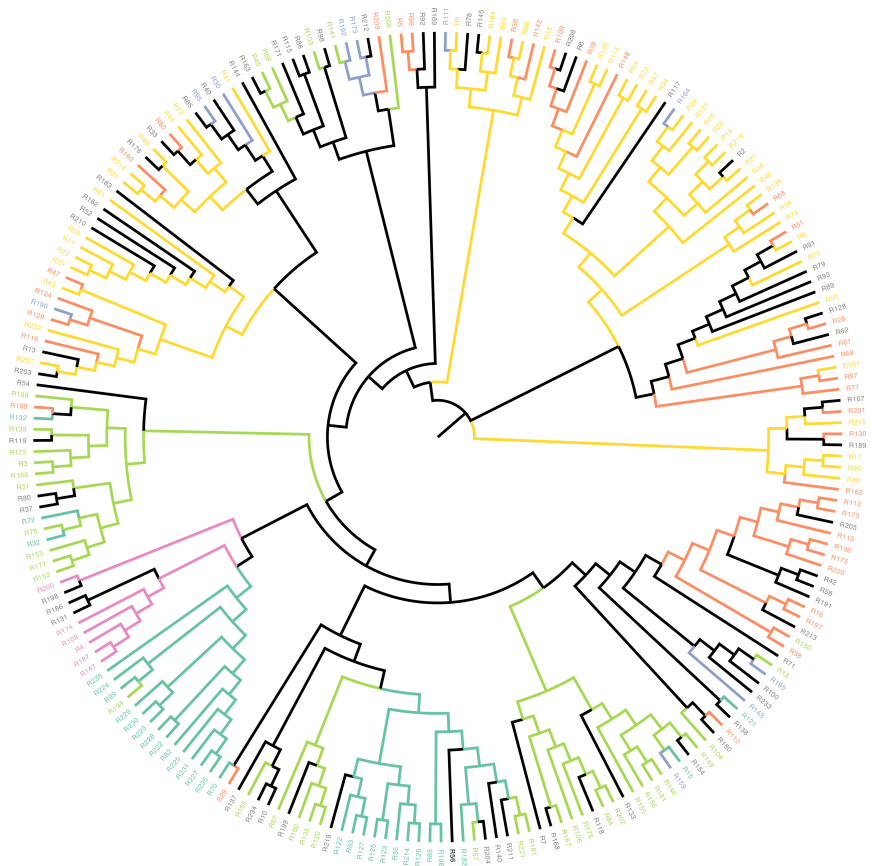


**Figure S1.** Neighbour-joining phylogenetic tree of 224 mango accessions based on SNPs. Branch colours indicate different subgroups of mango.


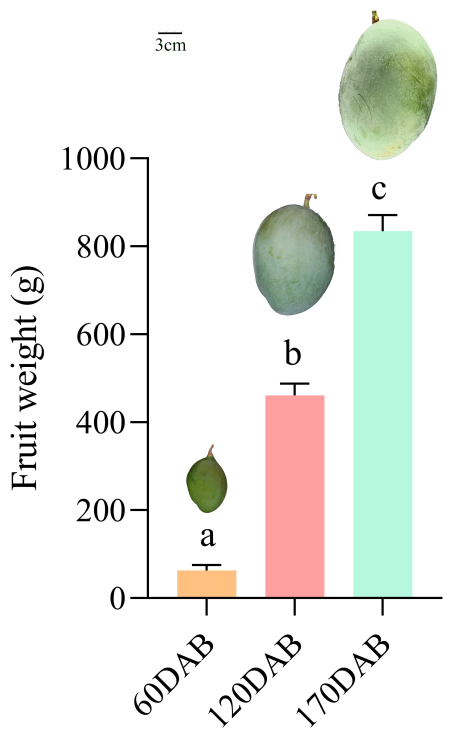


**Figure S2.** Characterization of ‘Keitt’ fruit growth and development. Phenotypic changes in fruit size and fruit weight at three developmental stages. Different letters stand for statistically significant differences in different stages calculated through one-way ANOVA (*P* < 0.05). DAB, Days after bloom.


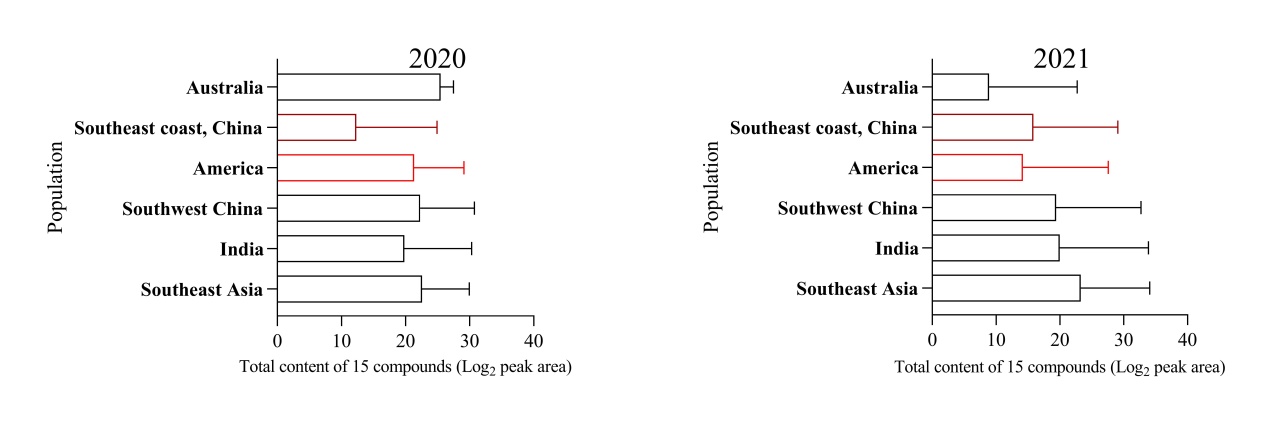


**Figure S3.** Total content of 15 aromatic compounds in different populations in 2020 and 2021.


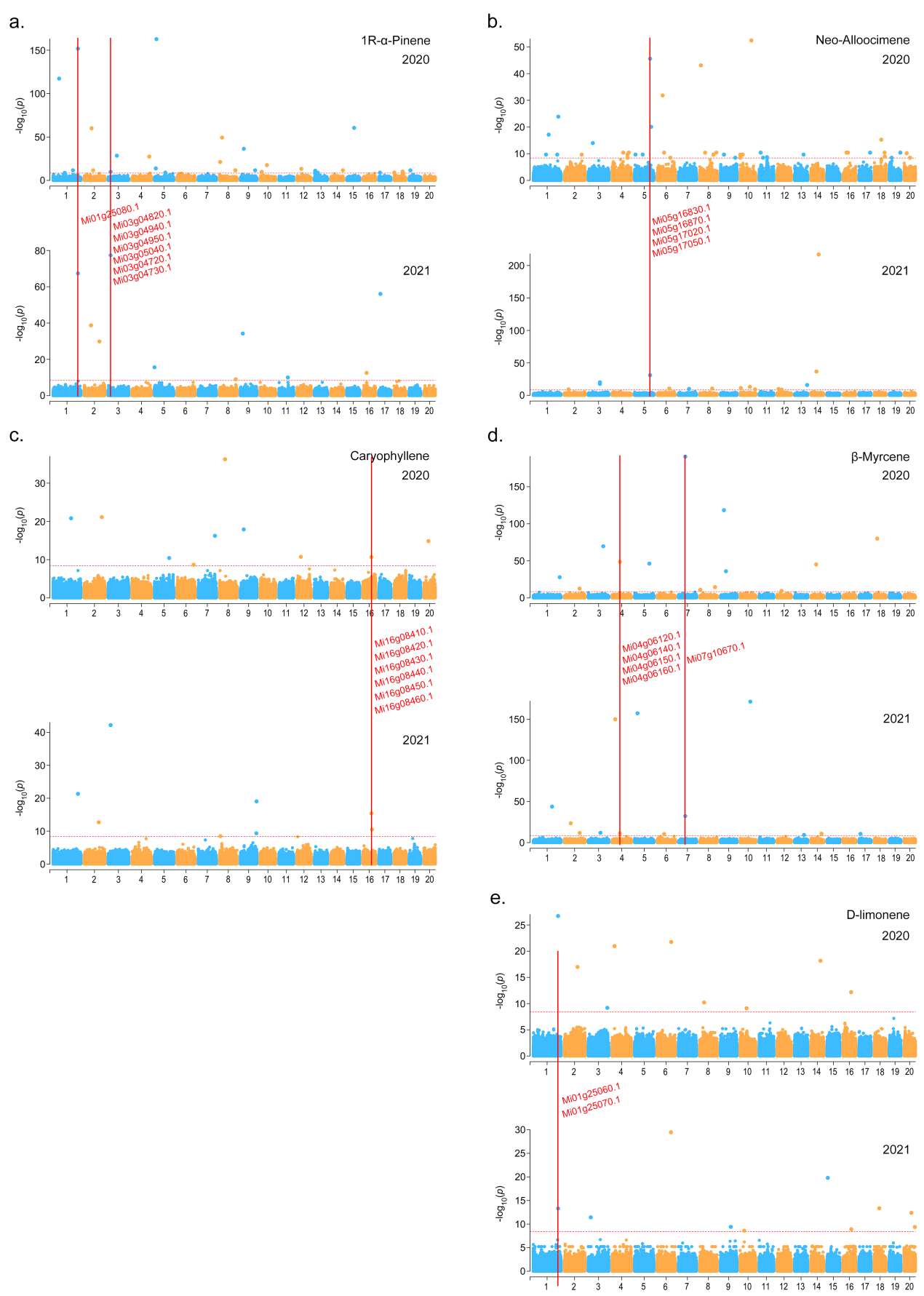


**Figure S4.** Manhattan plot of GWAS for 1R-α-Pinene (a), Neo-Alloocimene (b), Caryophyllene (c), β-Myrcene (d), and D-Limonene (e).


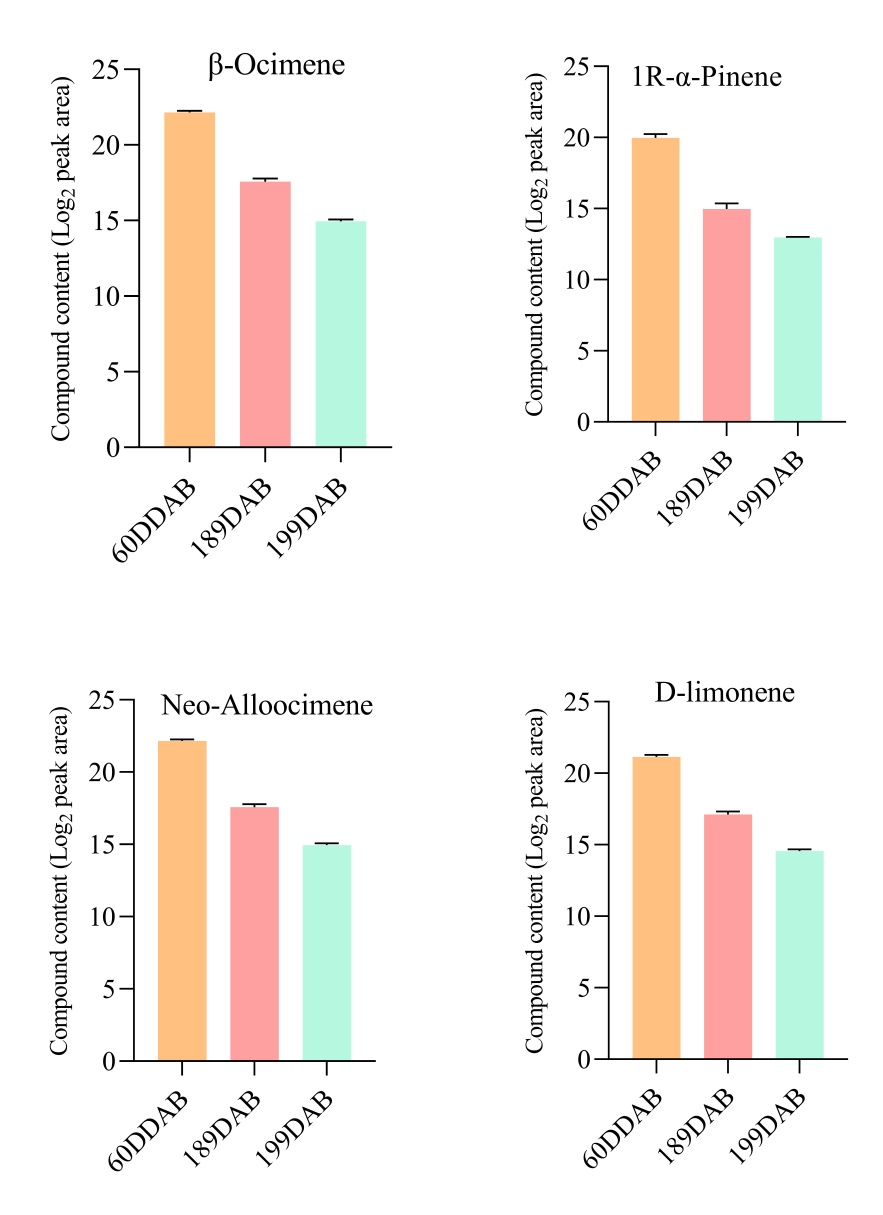


**Figure S5.** The accumulation pattern of β-Ocimene, 1R-α-Pinene, Neo-alloocimene, D-limonene, and at 60 DAB, 189 DAB and 199 DAB in mango fruit flesh. DAB, Days after bloom.
