# Supplementary figures and images for: Genomic diversity, population structure, and genome-wide association reveal genetic differentiation and trait improvements in mango
Source: Hortic Res. 2024 Jul 1;11(7):uhae153. doi: 10.1093/hr/uhae153 (PMC11246242; doi:10.1093/hr/uhae153)

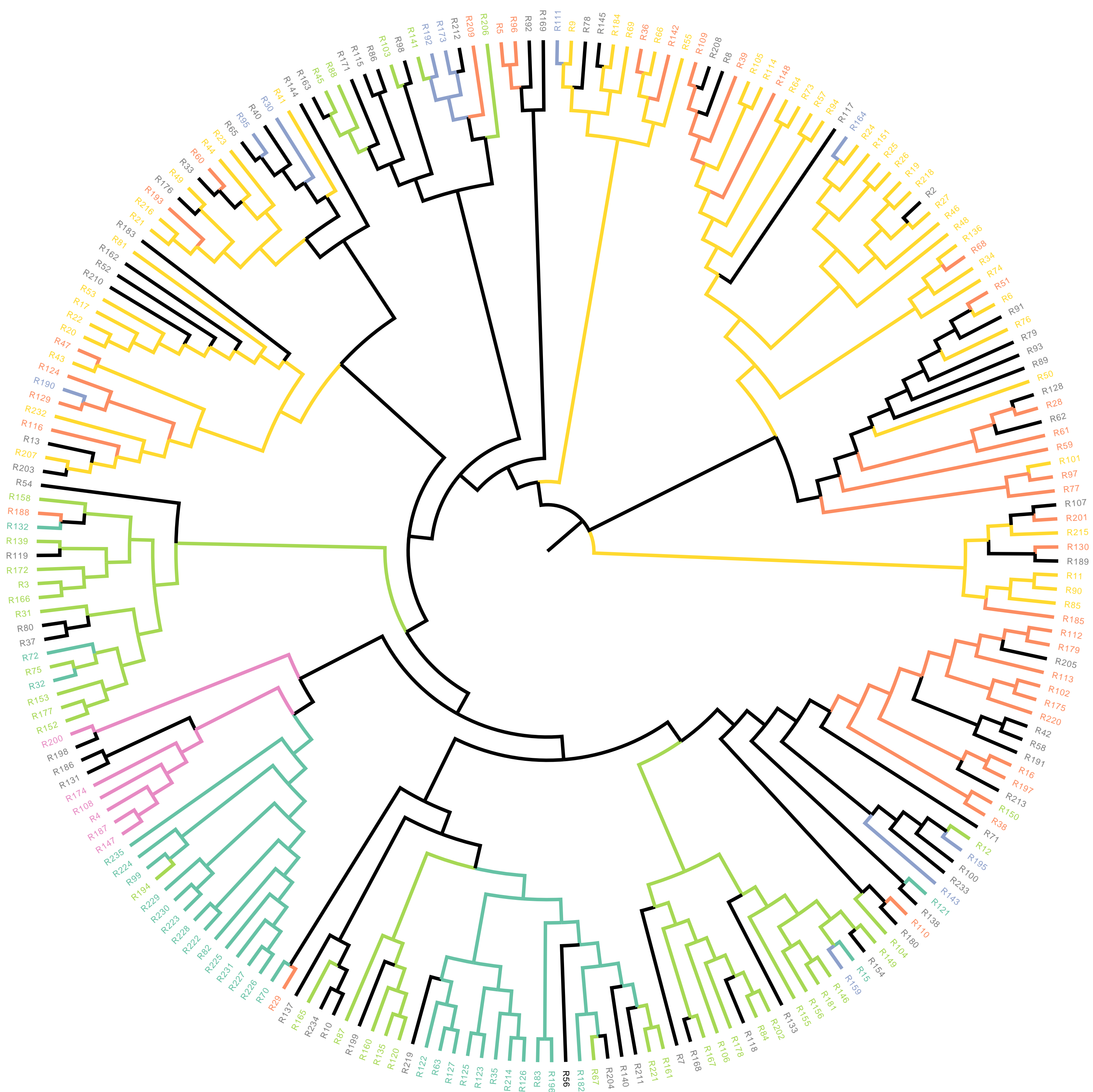

Supplement: Web_Material_uhae153 [file web_material_uhae153.zip › SuppFigure S1.pdf]

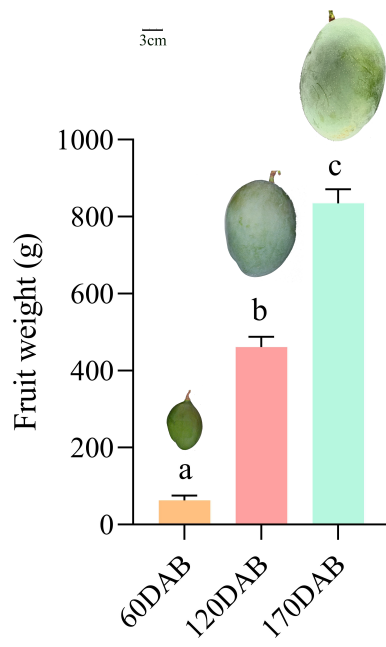

Supplement: Web_Material_uhae153 [file web_material_uhae153.zip › SuppFigure S2.pdf]

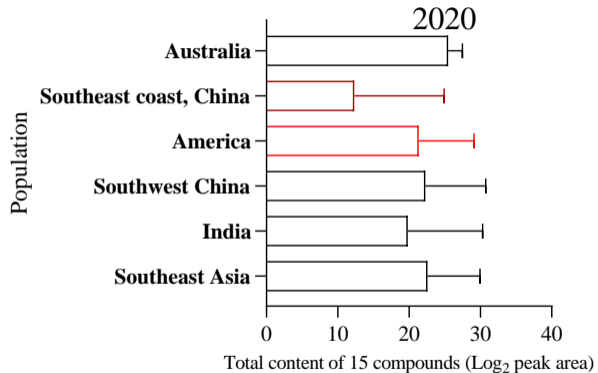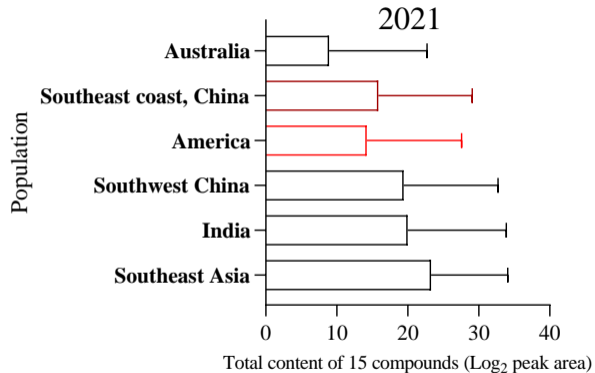

Supplement: Web_Material_uhae153 [file web_material_uhae153.zip › SuppFigure S3.pdf]

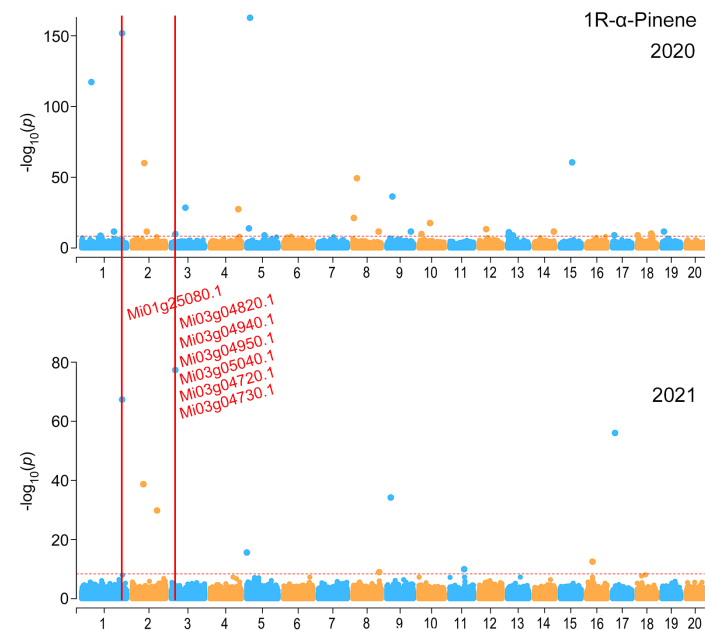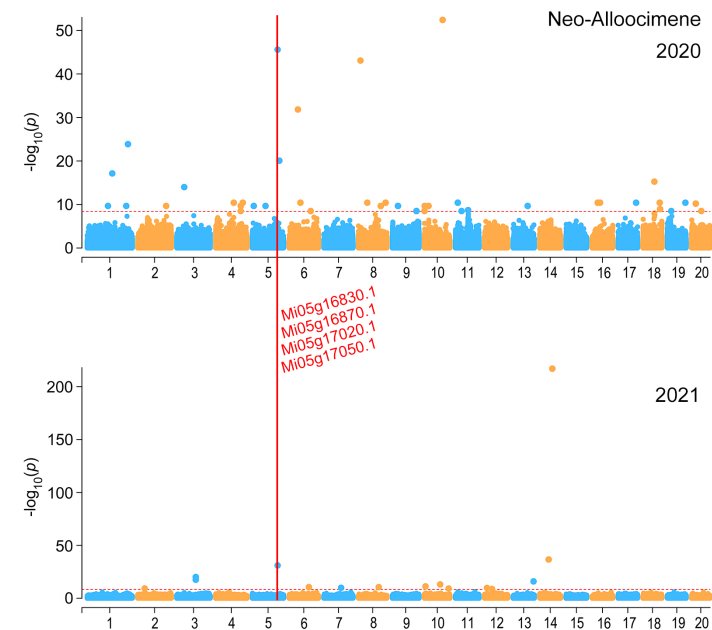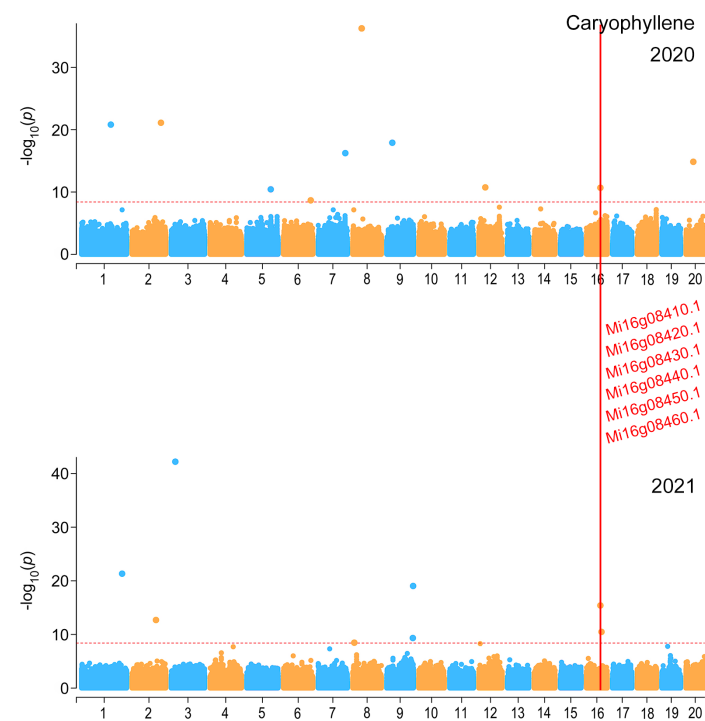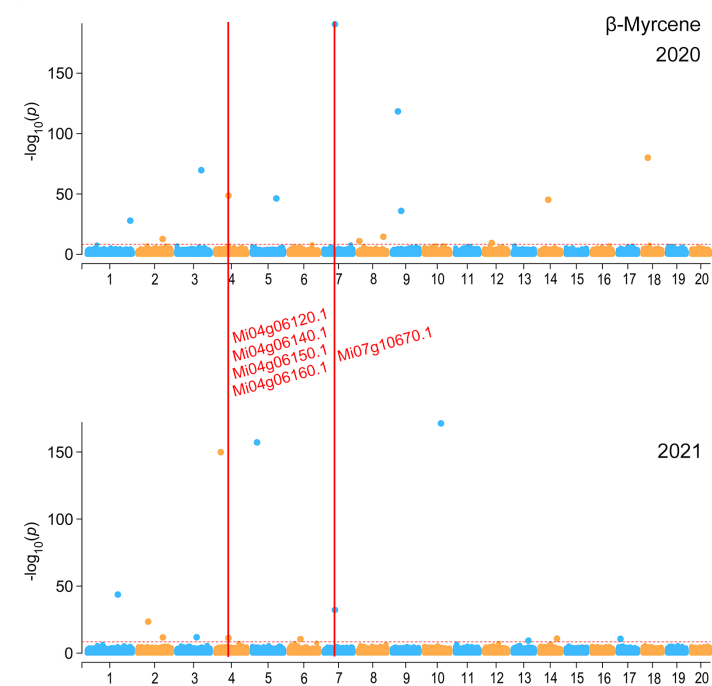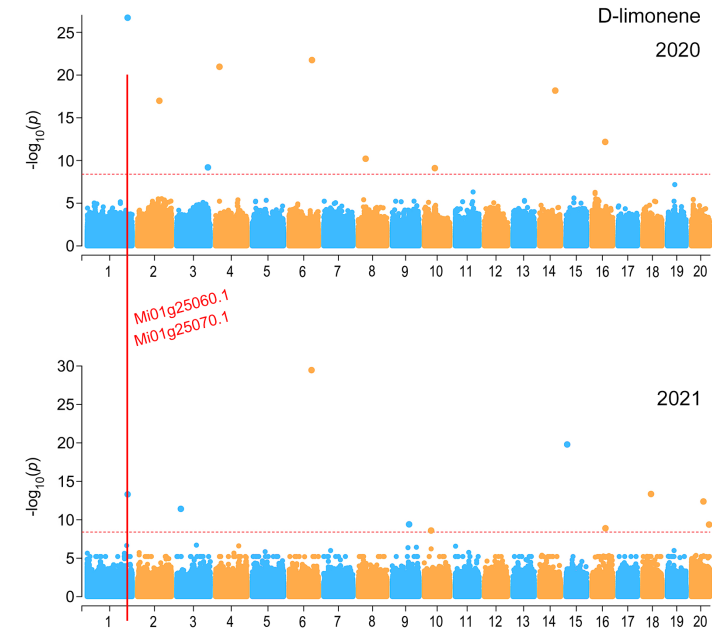

Supplement: Web_Material_uhae153 [file web_material_uhae153.zip › SuppFigure S4.pdf]

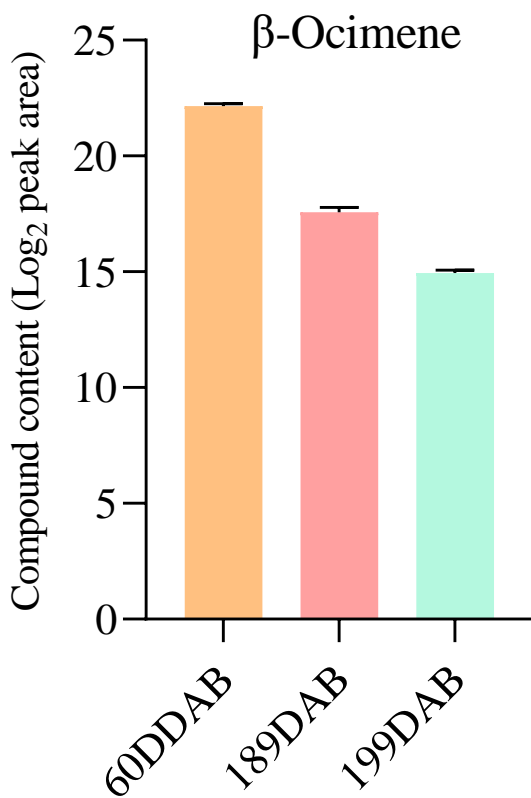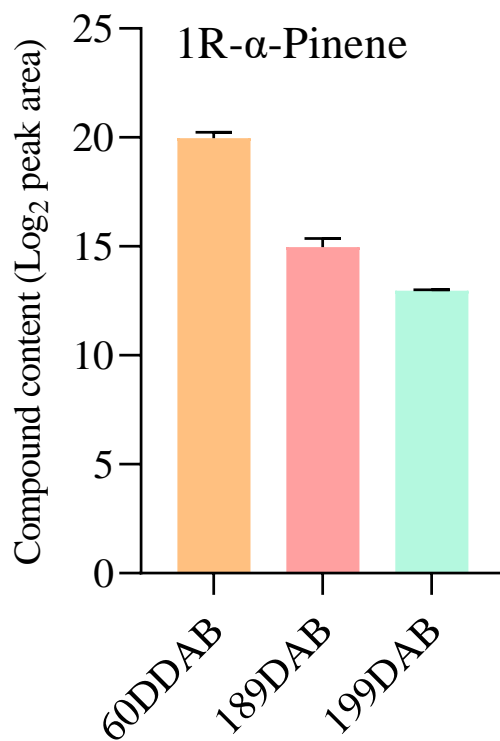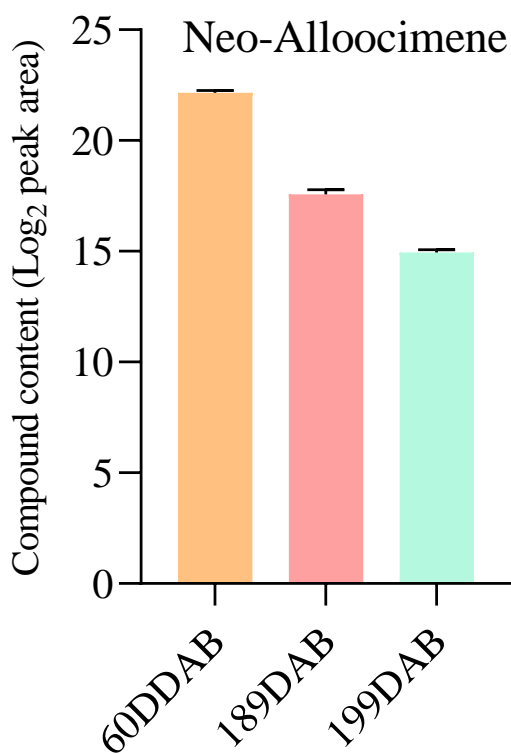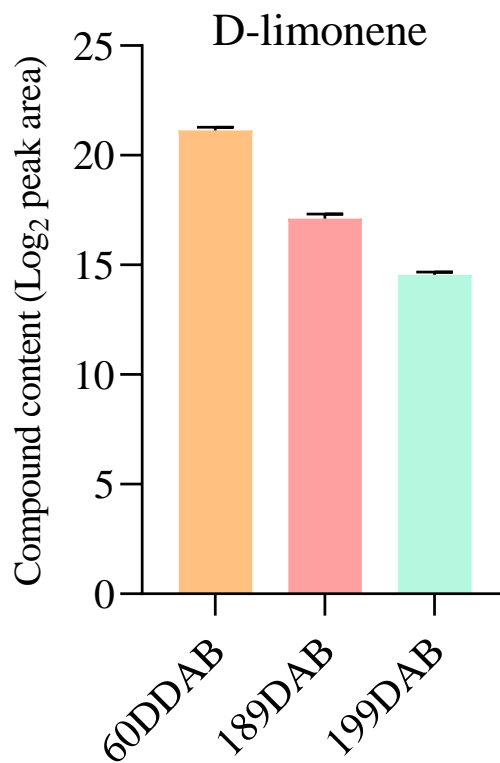

Supplement: Web_Material_uhae153 [file web_material_uhae153.zip › SuppFigure S5.pdf]
